# Comparison of a Novel Machine Learning–Based Clinical Query Platform With Traditional Guideline Searches for Hospital Emergencies: Prospective Pilot Study of User Experience and Time Efficiency

**DOI:** 10.2196/52358

**Published:** 2025-02-25

**Authors:** Hamza Ejaz, Hon Lung Keith Tsui, Mehul Patel, Luis Rafael Ulloa Paredes, Ellen Knights, Shah Bakht Aftab, Christian Peter Subbe

**Affiliations:** 1 Ysbyty Gwynedd, Clinical School, Clinical Research Office, Bangor, United Kingdom; 2 Medwise.ai Ltd, Leeds, United Kingdom; 3 Russells Hall Hospital, Dudley, United Kingdom; ^4^North Wales Medical School, Bangor University, Brigantia Building, Bangor, LL57 2AS, United Kingdom, 44 7771922890

**Keywords:** artificial intelligence, machine learning, information search, emergency care, developing, testing, information retrieval, hospital care, training, clinical practice, clinical experience, user satisfaction, clinical impact, user group, users, study design, mobile phone

## Abstract

**ABSTRACT:**

**Background:**

Emergency and acute medicine doctors require easily accessible evidence-based information to safely manage a wide range of clinical presentations. The inability to find evidence-based local guidelines on the trust’s intranet leads to information retrieval from the World Wide Web. Artificial intelligence (AI) has the potential to make evidence-based information retrieval faster and easier.

**Objective:**

The aim of the study is to conduct a time-motion analysis, comparing cohorts of junior doctors using (1) an AI-supported search engine versus (2) the traditional hospital intranet. The study also aims to examine the impact of the AI-supported search engine on the duration of searches and workflow when seeking answers to clinical queries at the point of care.

**Methods:**

This pre- and postobservational study was conducted in 2 phases. In the first phase, clinical information searches by 10 doctors caring for acutely unwell patients in acute medicine were observed during 10 working days. Based on these findings and input from a focus group of 14 clinicians, an AI-supported, context-sensitive search engine was implemented. In the second phase, clinical practice was observed for 10 doctors for an additional 10 working days using the new search engine.

**Results:**

The hospital intranet group (n=10) had a median of 23 months of clinical experience, while the AI-supported search engine group (n=10) had a median of 54 months. Participants using the AI-supported engine conducted fewer searches. User satisfaction and query resolution rates were similar between the 2 phases. Searches with the AI-supported engine took 43 seconds longer on average. Clinicians rated the new app with a favorable Net Promoter Score of 20.

**Conclusions:**

We report a successful feasibility pilot of an AI-driven search engine for clinical guidelines. Further development of the engine including the incorporation of large language models might improve accuracy and speed. More research is required to establish clinical impact in different user groups. Focusing on new staff at beginning of their post might be the most suitable study design.

## Introduction

In making decisions about patient care, clinicians frequently are faced with queries and are often unable to retrieve answers to them in a timely fashion. A systematic review [1] estimates that the per-patient frequency of queries raised by clinicians ranges from 0.4 to 0.8 per patient and that about two-thirds of these queries are left unanswered. This picture has been fairly stable over time despite the broad availability of web-based evidence resources that can answer these questions. Unanswered questions may lead to suboptimal patient care decisions and are missed opportunities for timely learning and practice improvement.

Medwise.ai is a solution codeveloped with Betsi Cadwaladr University Health Board), which helps clinicians find answers quickly from local guidelines and provide seamless and just-in-time access to high-quality evidence in the context of patient care decision-making in the clinical environment. A previous study [2] has demonstrated that smartphone apps can increase the speed to access guidelines when compared to using desktop computers. However, access to guidelines does not equate to finding answers to clinical questions quickly and effectively at the point of care. The Medwise.ai platform can collate local clinical guidelines and break them down into chunks of content that can be retrieved using natural language processing and information retrieval technologies as bite-sized answers for clinician’s questions. We conducted a time-motion analysis, comparing cohorts of junior doctors using an artificial intelligence (AI)–supported search engine and the traditional hospital intranet, to examine the impact of the AI-supported search engine on participant time and workflow when seeking answers to clinical queries at the point of care. The secondary end points of our study were to assess whether effective question answering could lead to a better quality of work life and improve confidence in decision-making for the end user.

This study aimed (1) to gain user feedback for a novel context-specific proof-of-concept search engine and (2) to measure the time required to retrieve clinically relevant information with a novel engine compared to other search engines in the setting of hospital emergency care.

## Methods

### Study Design

We conducted a prospective direct pre- and postobservational pilot study in teams caring for medical emergency admissions examining the impact of access to Medwise.ai, a clinical query answering platform, versus information retrieval from guidelines saved on the hospital intranet using time-motion study methodology [3].

### Study Setting

The study took place at the Ysbyty Gwynedd, Bangor. The Ysbyty Gwynedd is a district general hospital in North Wales with 550 beds covering all major specialties, including a dialysis unit and a 13-bedded critical care unit. Clinicians from 2 units in the hospital were recruited; the acute medical unit has 23 beds and is complemented by a same day emergency care unit for low-risk admissions.

Both units receive direct admissions from primary care and work closely with the emergency department (ED) to manage acute medical presentations. It is important to note that the traditional boundaries between emergency care and acute medicine have become less distinct in recent years due to increasing hospital overcrowding in the United Kingdom. While historically, patients were first seen in the ED by emergency physicians and then referred to the Acute Medical Unit for care by acute and general physicians, many acute and general medical teams now also work within the ED to manage the flow of acute medical patients.

During the day shift (8 AM to 8:30 PM), the on-call team consists of 4 doctors in training (a newly qualiﬁed foundation year 1 doctor, 2 core medical trainees with 1‐4 years of experience, and 1 medical registrar with 4 or more years of clinical experience and membership of the Royal College of Physicians) and an on-call consultant with a full specialist qualiﬁcation. The sole task of the on-call team is the care of emergency admissions and emergencies of inpatients. Patients seen by doctors in training are subsequently reviewed by a consultant as part of the posttake ward round.

### Intervention

Medwise.ai is a proof-of-concept search engine combining well-established information retrieval techniques with textual question answering and trained models that determine the best answer within a document. The search platform is available to the participating clinicians over a web app accessible via mobile web browser on both Android and iOS devices. Research staff trained the onboard clinicians and helped to install the Medwise.ai search on the participating clinicians’ mobile devices ahead of observations.

Content development for Medwise.ai was informed by informal interviews (Multimedia Appendix 1) with staff and a single focus group, consisting of 14 participants, to identify relevant local guidelines and standard operating procedure documents to be included in the Medwise.ai platform, thus ensuring user buy-in and optimal functionality of the Medwise.ai platform. The local documents shared with Medwise.ai were in PDF or Microsoft Word format. It is important to note that searches using Medwise.ai were limited by the content available in the local repository; if the content did not exist within this repository, the AI would not be able to provide answers to queries. It should be noted that the underlying AI model’s technical performance was not the focus of this study.

### Research Procedure

A dedicated and trained member of the research team shadowed trainee doctors for 20 complete working days: observation days included 10 days without and 10 days with access to Medwise.ai. The 2 groups of participants were not matched for the hospital intranet versus AI-supported search engine using groups.

Doctors reviewed new admissions and conducted unscheduled reviews of previously admitted patients: the doctors were aware that they were observed, and written consent was taken prior to commencing the direct observation. The observed activity of doctors was entered into a work diary. For practical reasons, all observations were undertaken during office hours.

### Sampling and Recruitment

The sample size was chosen pragmatically based on experience with previous time-motion studies [4]. Doctors working as part of the acute medical take throughout Ysbyty Gwynedd were recruited. To be included, participants had to be willing and able to give informed consent for participation in the study, be in possession of a smartphone to access web-based content, be permanent or locum staff, and be qualified as a doctor of any grade. Participants had the right to withdraw from the study at any time. Participants received vouchers with a value of US $50 for participation in the study. Doctors who were only part of the clinical team for 4 hours or less were excluded from recruitment.

Candidates were approached by the principal investigator (CPS) or the dedicated research team member. Notification of the study was undertaken through existing WhatsApp groups and a Junior Doctors’ Forum. Inclusion and exclusion criteria were identified during the initial screening.

### Assessments

Participants’ gender, specialty, grade, duration of work in their current post, and duration of clinical practice were recorded. Participants were shadowed for the duration of a shift but for a minimum of 6 hours per study day. Shadowing did not include times when doctors were consulting or examining patients.

Task duration for clinical queries was measured in minutes. Tasks were classified using a standardized list. Observed searches for information were classified by clinical content (diagnostic category, diagnostic pathway, treatment protocols, prognostication, scoring systems, normal values, and accessed data source). Confidence in decision-making was assessed at the end of each working day using a validated scale for self-assessment [5]. Confidence in decision-making and user satisfaction were measured using the Likert scale (How satisfied are you with the Medwise.ai product? On a Likert scale of 1-10) and the Net Promoter Score (NPS) [6] (How likely are you to recommend Medwise.ai to a friend or colleague?). NPS is a validated score that is used to measure user satisfaction. It is widely used in industry as a benchmark for comparing products within different industries. A positive NPS is considered as good, a score above 20 as favorable, and above 50 as excellent. Participants were also asked for suggestions for improvements of the platform.

### Data Management and Statistics

Statistical analysis was primarily descriptive including key data items related to the frequency of clinical searches, the subject of such searches, the time taken for the searches, and graded feedback in relation to the app. Data were recorded on paper case report forms and subsequently transcribed into a Microsoft Excel database for further analysis. Data were anonymized through the removal of name, case report number, and age.

The comparison was made between the events during 10 observed shifts without the app and 10 observed shifts with the app. Diary data and data from questionnaires were analyzed using SPSS (IBM Corp). Kernel density plots were used to visualize the differences in observed task duration; Welch *t* test (2-tailed) was used to determine whether the apparent differences in distribution were likely to have occurred due to random chance or were due to a real difference in the mean task duration.

Given the sample size and the unknown baseline distribution of the variables in question, statistical comparison data from this study might inform subsequent power calculations for related research or a definitive trial.

### Ethical Considerations

National Health Service (NHS) Research Ethics Review was not required as the study involved staff only as participants. The study was approved through Health and Care Research Wales and the Health Research Authority. No adverse events were anticipated. However, monitoring was in place in line with the sponsor safety reporting standard operating procedure. Data from focus group meetings were anonymized and deidentified. Written informed consent was given by all members of staff who were observed during the assessments. Participants were reimbursed with £50 (US $61.92) retail vouchers according to local regulations.

## Results

### Demographics and Baseline Characteristics

All assessments were undertaken between November 17, 2022, and February 8, 2023 (hospital intranet period) and July 6 and 27, 2023 (AI-supported search engine period).

The hospital intranet and AI-supported search engine groups both comprised 10 doctors each with 5 and 7 female doctors, respectively. The hospital intranet group had a median clinical experience of 23 months. Of these, 6.1 months were in their current specialty. The AI-supported search engine group had a median of 54 months of experience. Of these, 9.6 months were in their current specialty. No statistically significant differences between mean time in current specialty were observed between the groups (*t*=−1.01; 95% CI −11.4679 to 4.0179; *P*>.05).

### Focus Group

The focus group was held on April 5, 2023, after the completion of the hospital intranet period. In total, 14 clinicians participated including foundation year 1 and 2 doctors, registrars, physician associates, and 3 consultant physicians. Participants were asked a list of prepared questions in relation to search habits, preferred information sources, and views about search engines (Multimedia Appendix 1).

Contributors discussed sources of information that they use for their clinical practice and recommended examples of web resources from other NHS organizations. Pros and cons of alternative models of information provision including share point sites were debated. Clinicians commented on the difficulties to find the right locally authorized resources in locations of the internet and intranet. On the other hand, they valued that Medwise.ai guidance is relevant for the organization in which the search is undertaken. Participants discussed their experience of using resources from other UK and international organizations if there is no identifiable local guidance for a topic. Doctors were concerned that Google searches might identify sources that contain misinformation. The challenge of identifying the right information in real time was described as crucial during the time pressures of acute and emergency care.

### Descriptives

Overall, 67 searches were observed in the hospital intranet and 39 searches in the AI-supported search engine period. The mean number of clinical searches performed per shift was 6.7 (SD 3.43) in the hospital intranet group and 3.9 (SD 3.00) in the AI-supported search engine group. Mobile phones were used for 40 of 67 (60%) searches in the hospital intranet group and 34 of 39 (85%) in the AI-supported search engine group. In the hospital intranet group, 11 searches were via Google. Other sources included apps, the British National Formulary, National Institute for Health and Care Excellence guidelines, and local guidelines. In the AI-supported search engine group, no search required Google.

Searches covered a large number of topics (MultimediaAppendices 2  3). The most commonly searched topics in both groups were medication-related (n=18 and n=10, respectively).

Participants in the AI-supported search engine group commented on the impact of Medwise.ai on workflow using free-text responses (Textbox 1).

Textbox 1. **Free-text feedback provided by users collected at the end of observation.**

“With more training [training of the AI system] this could be very helpful in speeding up carrying out jobs. Bit unfortunate study was carried out in final weeks of rotation, when doctors are the most comfortable with systems and need to search things very rarely” [Participant 1].

“Can see it has the potential to increase speed of doing jobs, thus means we see patients quicker” [Participant 2].

“May expediate time available for patient care if brings up more precise answers. When I know where to find the answers it is easier to go straight to the source” [Participant 3].

“As you get your answers under one second from hospital protocol, it saves time and helps you see more patients. also can provide more time for patient care rather than looking for protocol and guidelines in each app, it will be all in one. Highly recommended” [Participant 4].

“Probably not much difference. Perhaps better used in GP. I think it would be more useful in GP. In hospital it is okay but not majorly useful. I think over all it would be a good idea. But I think I search for stuff more in GP. I think overall ED staff would use it most in hospital” [Participant 5].

“Easy availability of resources saves up time and causes less disruption to the flow of clerking. Easy access to BCU/local guidelines is very helpful as some of these are difficult to find on the intranet. Great idea; would be really helpful” [Participant 6].

“Will reduce search time for searching guidelines resulting in more time for patient care. And all in one place makes it easy to find [information]. I am surprised that I have not needed to access my phone/ the app as yet today as this is unusual. Would usually look at betsinet multiple times during a shift for electrolyte levels. I anticipate that this app would be useful” [Participant 7].

“Makes it easier to search for relevant guidelines/policies/pathways etc. Easy and quick to use. User friendly. Appears to work well for searches during work time. Quickly found everything I searched for” [Participant 8].

“Will help find relevant guidelines much more quickly, which will make work more efficient” [Participant 9].

“Directed me to mdcalc which I already use as an app. No impact on above criteria (workflow, number of patients seen and time for each patient). Would be more useful to have easy access to trust guidelines such as electrolyte imbalances and chest pain etc.” [Participant 10].

### Comparison of Groups

**Figure 1.**
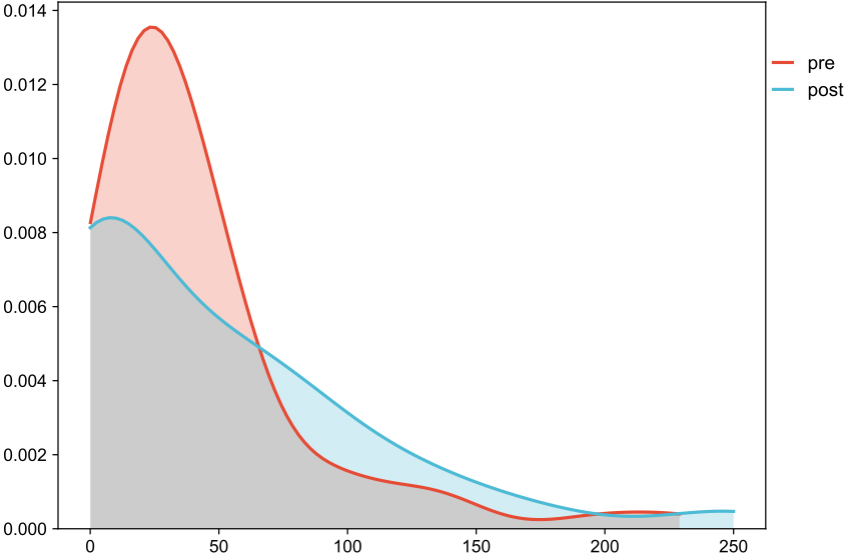
Kernel plot of the duration of searches pre (hospital intranet) and post (artificial intelligence–supported search engine) phase.

Comparison of the 2 groups revealed that searches in the hospital intranet group had shorter search times than in the AI-supported search engine group (41.4 vs 88.1 seconds, respectively; Welch *t*=4.06; 95% CI 20.30-59.09; *P*<0.05). The differences in the observed task duration are presented as Kernel density plots in Figure 1. There were no statistically significant differences between the groups with regard to user satisfaction (Welch *t*=0.75; 95% CI −1.2607 to 2.6607; *P*>.05) and likelihood of a search result solving the query (*χ*^2^=2.2; 95% CI −3.24% to 27.45%; *P*>.05). Participants of the AI-supported search engine group were likely to recommend Medwise.ai to a friend or colleague as reflected in their NPS of 20 (40% promoters and 20% detractors).

Overall, the group using Medwise.ai spent longer searching for information, and their searches were not more likely to be successful compared to the usual information retrieval methods.

## Discussion

### What We Have Shown

To the best of our knowledge, this is the first study to evaluate the impact of an AI-based information retrieval system on clinical workflow in acute hospital care. The design and implementation of a clinical information search engine integrating intranet- and internet-based information resources using machine learning was feasible and well-received by participants.

Searches in this small sample were not faster, and there was no higher likelihood of a successful search result. Despite this, doctors believe Medwise.ai has the potential to improve efficiency and workflow in the future and hence highly recommended the digital tool to their colleagues.

### Strength and Weaknesses

One of the strengths of our study is that it uses objective measures of efficiency including search times and search outcomes rather than relying on self-reported questionnaire data from users. This mitigates recall bias. Additionally, the free-text feedback from users provided useful insights in that it shows that despite Medwise.ai’s limitations, the users were optimistic about its potential in the future and expressed views in favor of its implementation in clinical workflow, as evident in quotes in Textbox 1.

It is worth noting that no specific training in evidence searching was provided to the participants beyond what they would have received as part of their usual medical education. This lack of additional training could be seen as both a strength and a limitation of our study. On one hand, it reflects the real-world scenario where clinicians often rely on their existing search skills when using new tools. This approach allows for a more authentic assessment of the AI-supported search engine’s usability and effectiveness in a clinical setting. On the other hand, it raises questions about whether targeted training in evidence searching or in using the new platform might have improved search efficiency and outcomes. The ideal search engine should require minimal introduction or training, but understanding its functionality could potentially enhance search yields. Future studies might consider comparing the performance of users with and without specific search training to better understand the impact of such preparation on the effectiveness of AI-supported search tools in clinical practice.

The initially unfamiliar user interface could have been a significant contributor to the longer search time. Additionally, the introduction of the AI-supported search engine occurred toward the end of the rotation when doctors were most confident about the processes and management of patients. Participants had spent an average of 8 months in their current specialty, throughout which they would have regularly used traditional information retrieval sources.

Our observations and user feedback highlighted several areas for potential improvement in future iterations of the AI-supported search engine. First, we recognized that the platform was initially developed for desktop use, but mobile access emerged as the preferred method in the acute care setting. In response, the platform’s developers are working on a mobile-first version, including dedicated iOS and Android apps, as well as access via a chatbot interface on WhatsApp. This adaptation aims to improve user experience and reduce search times. Additionally, we noted that the more experienced intervention group might have been less accustomed to new digital formats, potentially impacting their interaction with the novel interface. To address this, future iterations could incorporate more intuitive design elements and provide brief, targeted training to familiarize users with the system’s capabilities.

In our pilot, Medwise.ai was neither more nor less efficient, successful, or satisfying to users than traditional information retrieval sources. Searches using Medwise.ai were limited by the content available: if content did not exist within the local repository, then the AI will not be able to provide answers to queries. Finally, the local documents that were shared with Medwise.ai were in PDF or Microsoft Word format. Further development to transform these documents into more machine-readable formats with seamless integration with the search could further improve the performance of the Medwise.ai search engine. Like all AI-based tools, Medwise.ai’s performance is contingent upon the quality and quantity of data used to train the tool. As the bank of searches builds up over time, Medwise.ai would be able to offer easier and faster access to reliable local guidelines.

The effectiveness of the search engine is dependent on the underlying AI models’ performance. Although not a focus of this study, it is an area for future attention, particularly with the growing use of large language models.

### What Others Have Shown

The NHS Long Term Workforce Plan [7] emphasizes the need to leverage the power of AI to improve efficiency and workflow. This case study is an example of a technological innovation to improve work efficiency with the end goal of improving patient care. It contributes to the literature focusing on evaluating the potential of AI-based technologies to improve service provision in the NHS [8-10].

### Clinical Implications

The search engine was implemented successfully during this trial. With the clear hierarchy of information sources, Medwise.ai might represent a source that has higher utility for clinicians at the bedside than public search engines such as Google or Safari. While it has been hypothesized that AI could aid to bring clinical guidelines to routine consultations [11], clinical apps remain scarce [12]. Moreover, evidence suggesting a significant reliance on colleagues and internet websites for information retrieval during shifts makes a strong case for the need for easy-to-access evidence-based local guidelines [13].

### Research Recommendations

**Figure 2.**
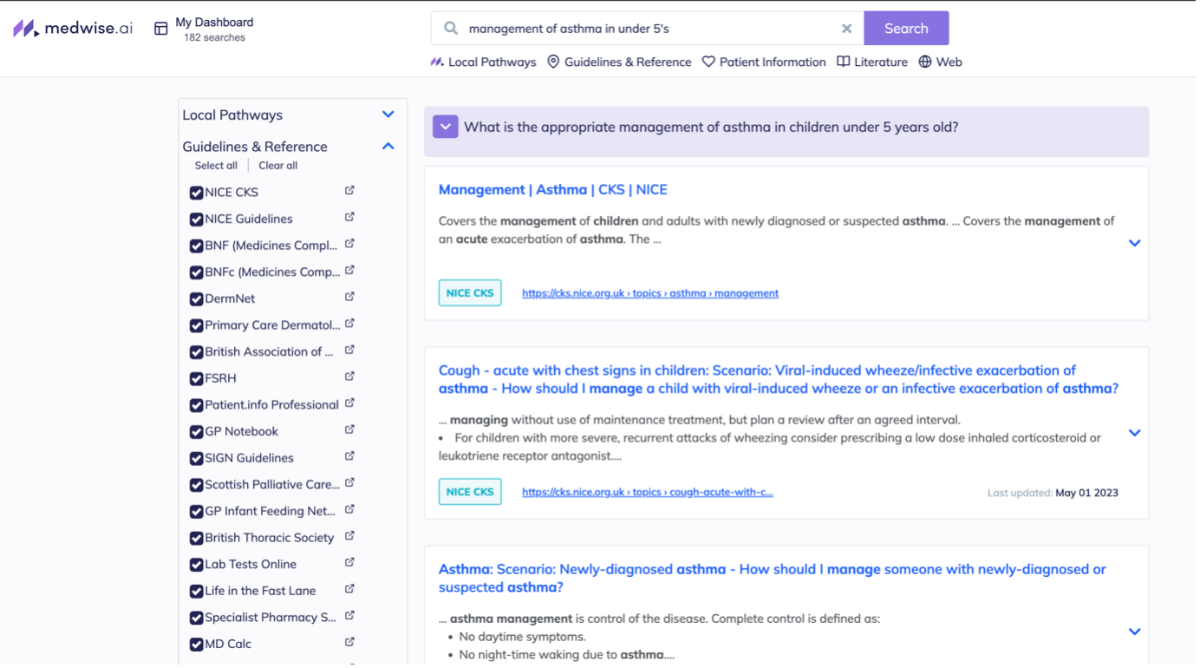
An image of the platform’s search reply.

Given the small sample size and the unknown baseline distribution of the variables in question, statistical comparison is expected to be of limited value. A larger study with randomization of doctors at the beginning of rotational posts might provide a more robust assessment. The authors believe that less experienced clinicians (foundation year 1 trainees or advanced nurse practitioners in their first year of practice) would rely heavily on digital information sources and would be equally unfamiliar with other information retrieval methods and might hence be the most suitable group to evaluate effects on decision-making, safety, and possible clinical impact. More recent developments in AI such as large language models have shown the ability to encode clinical knowledge and answer medical questions [14]. Integrating these into the Medwise.ai engine might improve accuracy and speed. In the proof-of-concept platform, a full-text, query-augmented, retrieval engine configured and tuned for medical searches was used (Figure 2). No large language models were used. Further research is needed to evaluate how the accuracy and performance of the proof-of-concept search engine could be improved by incorporating large language models into the engine.

### Conclusions

Implementation of Medwise.ai was feasible. In this small pilot study of an AI-supported search engine, we did not demonstrate increased workflow efficiency, search success, or satisfaction when compared with searches using the traditional hospital intranet. However, doctors in training believed the solution has the potential to do so in the future and hence recommend its implementation in clinical workflow. With added source content and integration of the Medwise.ai search with the next generation of AI large language models, it is likely that more benefits will be realized.

## Supplementary material

10.2196/52358 Multimedia Appendix 1 Questions asked in the focus group.

10.2196/52358 Multimedia Appendix 2 Clustered column chart showing the distribution of search categories in the hospital intranet period (control) and artificial intelligence–supported search engine period (intervention). “General calc” comprised of calculating medication doses and converting units of measurements. “Interpreting invx” meant finding information about the significance of specific findings on investigations including plain films, electrocardiographs, and blood results. “Medication” meant medication-related information, that is, dose, route of administration, and interactions. “Scoring systems” meant users calculating prognostic scores including Well score. “Staff contact“ meant looking up bleep numbers for other members of the health care workforce. “Term” meant looking up specific definitions. “Treatment protocol” meant treatment pathways for conditions and presenting complaints.

10.2196/52358 Multimedia Appendix 3 Word cloud of search terms.

## Abbreviations

AI: artificial intelligence
ED: emergency department
NHS: National Health Service
NPS: Net Promoter Score
