# Supplementary material for: Comparison of a Novel Machine Learning–Based Clinical Query Platform With Traditional Guideline Searches for Hospital Emergencies: Prospective Pilot Study of User Experience and Time Efficiency
Source: JMIR Hum Factors. 2025 Feb 25;12:e52358. doi: 10.2196/52358 (PMC11878475; doi:10.2196/52358)
Supplement: Multimedia Appendix 1 [file humanfactors-v12-e52358-s001.docx]

Which resources do you use to retrieve information during your shift?

How reliable do you find web-based resources?

What are the biggest hurdles when retrieving guidelines from the local intranet?
