# Supplementary figures and images for: Comparison of a Novel Machine Learning–Based Clinical Query Platform With Traditional Guideline Searches for Hospital Emergencies: Prospective Pilot Study of User Experience and Time Efficiency
Source: JMIR Hum Factors. 2025 Feb 25;12:e52358. doi: 10.2196/52358 (PMC11878475; doi:10.2196/52358)

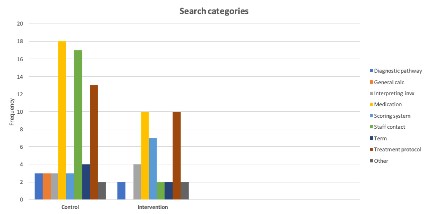

Supplement: Multimedia Appendix 2 [file humanfactors-v12-e52358-s002.jpeg]

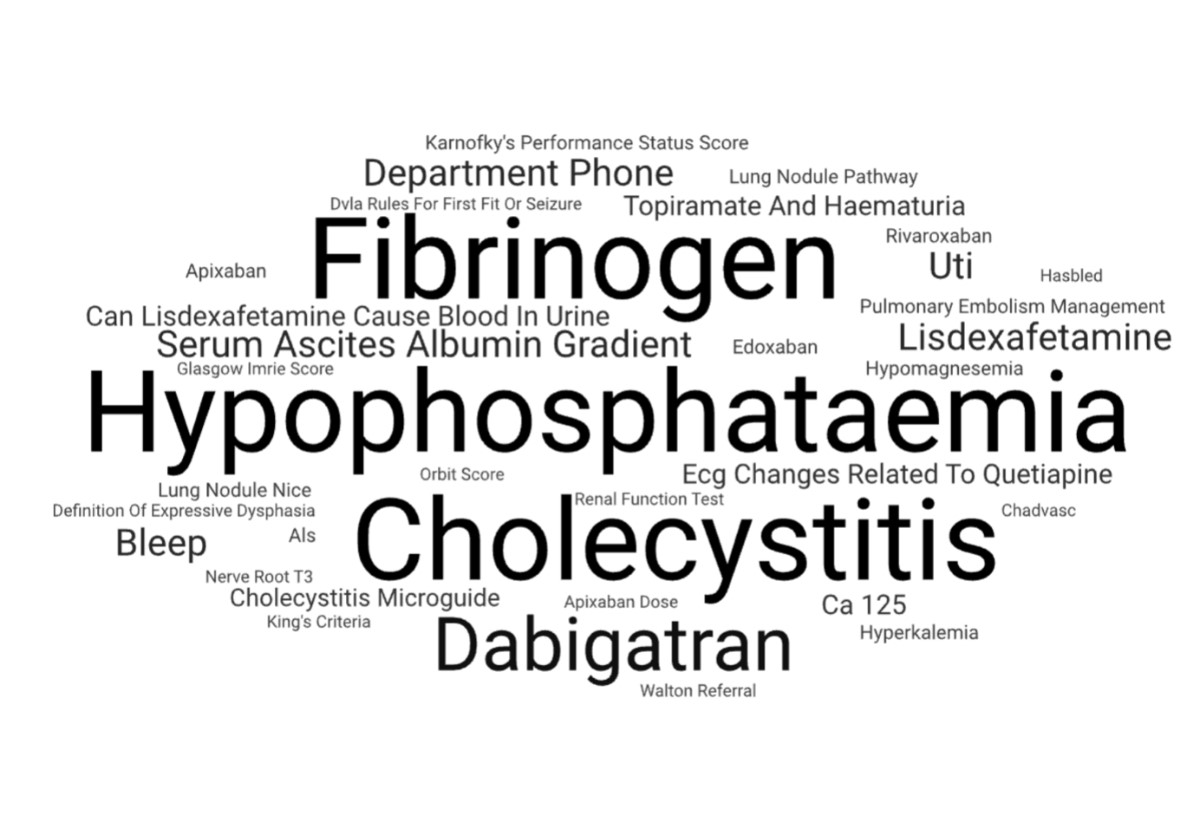

Supplement: Multimedia Appendix 3 [file humanfactors-v12-e52358-s003.jpeg]
